# Pathogenesis of acephalic spermatozoa syndrome caused by *PMFBP1* mutation

**DOI:** 10.1186/s12610-024-00240-3

**Published:** 2024-12-13

**Authors:** Huaqiang Xia, Juan Zhang, Wuyuan Mao, Kangle Yi, Teng Wang, Lingyan Liao

**Affiliations:** 1 Reproductive Medicine Center, Zhuzhou Central Hospital, Zhuzhou Hospital Affiliated to Xiangya School of Medicine, Central South University, Zhuzhou, Hunan 410120 China; 2 Hunan Institute of Animal and Veterinary Science, Changsha, 41000 Hunan China; 3 Pharmacy Department, Zhuzhou Central Hospital, Zhuzhou Hospital Affiliated to Xiangya School of Medicine, Central South University, Zhuzhou, Hunan 410120 China

**Keywords:** Acephalic spermatozoa syndrome, Polyamine-modulated factor 1 binding protein 1 (*PMFBP1*), Splice-site mutation

## Abstract

**Background:**

Acephalic spermatozoa syndrome is a rare but severe type of teratozoospermia. The familial trait of acephalic spermatozoa syndrome suggests that genetic factors play an important role. However, known mutations account for only some acephalic spermatozoa syndrome patients, and more studies are needed to elucidate its pathogenesis. The current study aimed to elucidate the pathogenesis of acephalic spermatozoa syndrome caused by *PMFBP1* mutation.

**Results:**

We identified a homozygous splice site mutation (NM_031293.2, c.2089-1G > T) in *PMFBP1* through Sanger sequencing. Western blotting and immunofluorescence analyses revealed that this splice site mutation resulted in the absence of *PMFBP1* protein expression in the patient's sperm cells. We generated an in vitro model carrying the splice site mutation in *PMFBP1* and confirmed, through RT‒PCR and Sanger sequencing, that it led to a deletion of 4 base pairs from exon 15.

**Conclusion:**

A homozygous splice site mutation results in a deletion of 4 bp from exon 15 of *PMFBP1*, thereby affecting the expression of the *PMFBP1* protein. The absence of *PMFBP1* protein expression can lead to acephalic spermatozoa syndrome. This finding elucidates the underlying cause of acephalic spermatozoa syndrome associated with this specific mutation (NM_031293.2, c.2089-1G > T) in *PMFBP1*.

## Résumé

**Contexte:**

Le syndrome des spermatozoïdes acéphaliques est un type rare mais grave de tératozoospermie. Le caractère familial du syndrome des spermatozoïdes acéphaliques suggère que les facteurs génétiques jouent un rôle important. Cependant, les mutations connues ne représentent que quelques patients atteints du syndrome des spermatozoïdes acéphaliques, et d’autres études sont nécessaires pour en élucider la pathogenèse. La présente étude visait à élucider la pathogenèse du syndrome des spermatozoïdes acéphales causé par la mutation PMFBP1.

**Résultats:**

Nous avons identifié une mutation homozygote du site d’épissage (NM_031293.2, c.2089-1G > T) dans PMFBP1 par séquençage Sanger. Les analyses par Western blot et par immunofluorescence ont révélé que cette mutation du site d’épissage entraînait l’absence d’expression de la protéine PMFBP1 dans les spermatozoïdes du patient. Nous avons généré un modèle in vitro portant la mutation du site d’épissage dans PMFBP1 et avons confirmé, par RT-PCR et séquençage Sanger, qu’elle conduisait à une délétion de 4 paires de bases de l’exon 15.

**Conclusions:**

Une mutation homozygote du site d’épissage entraîne une délétion de 4 paires de bases de l’exon 15 de PMFBP1, affectant ainsi l’expression de la protéine PMFBP1. L’absence d’expression de la protéine PMFBP1 peut entraîner un syndrome des spermatozoïdes acéphales. Cette découverte élucide la cause sous-jacente du syndrome des spermatozoïdes acéphaliques associée à cette mutation spécifique (NM_031293.2, c.2089-1G > T) dans PMFBP1.

**Mots-clés:**

Syndrome des Spermatozoïdes acéphaliques; Protéine de Liaison 1 du Facteur 1 modulé par les Polyamines (PMFBP1); Mutation du Site d’Epissage.

## Introduction

Infertility is a global issue that affects approximately 15% of couples worldwide. Importantly, male factors contribute to nearly half of all infertility cases, highlighting the importance of addressing male infertility alongside female infertility [1, 2]. One common cause of male infertility is teratozoospermia, which refers to morphological defects in spermatozoa. Among the various types of teratozoospermia, acephalic spermatozoa syndrome (ASS) is rare yet severe [3–9]. This condition is characterized by the absence of the cephalic region in spermatozoa [5]. During ejaculation, patients with ASS typically exhibit a paucity of normal spermatozoa in their seminal fluid. Instead, they frequently present an abundance of headless sperm tails (pinhead sperm), a limited quantity of tailless sperm heads, and aberrant connections between the head and tail segments. These anomalous sperm exhibit intact structures, such as the nucleus, acrosome, and midpiece; however, the alignment of the midpiece with the rest of the sperm is disrupted [5].

ASS is often associated with genetic factors. In recent years, researchers have made progress in identifying specific genes that play a role in ASS. Mutations in several genes, such as *PMFBP1*, *SUN5*, *TSGA10*, *BRDT*, *HOOK1*, *DNAH6*, *ACTRT1*, *SPATC1L* and *SPATA20*, have been identified as causative factors for some patients [10–23]. However, it is important to note that these mutations account for only a portion of individuals diagnosed with ASS. To gain further insights into the pathogenesis of this condition and potentially identify additional contributing factors or genetic variations involved in ASS development, more extensive studies are needed.

In this study, we aim to elucidate the pathogenesis of ASS caused by *PMFBP1* mutation. We identified a specific genetic mutation associated with ASS. A homozygous splice site mutation (NM_031293.2, c.2089-1G > T) was found in the *PMFBP1* gene. Further analysis via western blotting and immunofluorescence techniques revealed that this splice site mutation led to the absence of the *PMFBP1* protein in the patient's sperm cells. Additionally, a minigene construction of *PMFBP1* was generated, and its mRNA expression level was subsequently confirmed through in vitro studies. These tests confirmed that the splice-site mutation caused a deletion of 4 base pairs from exon 15 of the *PMFBP1* gene. Our findings explain the pathogenesis that *PMFBP1* mutation induced ASS.

## Materials and methods

### Patient information

A 32-year-old man with a history of primary infertility for more than 5 years whose parents were consanguineous sought to identify genetic factors at the Reproductive Medicine Center of Zhuzhou Hospital Affiliated to Xiangya School of Medicine, CSU, in June 2023. He also has a sister who has a normal baby. The patient's karyotype and Y chromosome microdeletion were examined. The bilateral testicular size, spermatic vein, and prostate were assessed via color ultrasound imaging. This study was approved by the Ethics Committee of Zhuzhou Hospital Affiliated to Xiangya School of Medicine, CSU, and written informed consent was obtained from the participant.

### Computer-assisted semen and sperm morphology analysis

The patient abstained from sexual activity for a period of 3 to 5 days prior to the extraction procedure. The patient's semen was analyzed three times at the same reproductive center, following the guidelines outlined in the “WHO laboratory manual for the examination and processing of human semen” (Sixth Edition) [24]. The following parameters were observed: semen volume, pH, sperm concentration, sperm motility, and proportion of abnormal spermatozoa. (Table 1). Sperm morphology was assessed via Papanicolaou staining according to the “WHO laboratory manual for the examination and processing of human semen” (Sixth Edition). The morphology of sperm is divided into head anomalies, PI anomalies and flagellar anomalies.

**Table 1:** Semen parameters of the patient with ASS

Proband	1st		2nd		3rd	
Volume (ml)	2.8		3.4		3.1	
pH	7.5		7.4		7.5	
Concentration (10^6^/ml)	5.2		6.5		10.4	
Motility B + C (%)^a^	7.2		7.6		8.2	
Sperm morphology(%)	Abnormal head–tail junction	2	Abnormal head–tail junction	3	Abnormal head–tail junction	2.3
	Decaudated	1	Decaudated	1	Decaudated	1.7
	Acephalic	97	Acephalic	96	Acephalic	96

^a^Motility B+C:B=spermatozoa with progressive motility and C= small circular motion or tail swing

### Sanger sequencing of PMFBP1 and SUN5

Patient family (patient, patient parents and his sister) peripheral blood was collected and stored at -80 °C in a refrigerator for preservation purposes. Genomic DNA extraction from the collected blood sample was conducted via the QIAamp DNA Blood Mini Kit (QIAGEN, Hilden, Germany) according to the manufacturer's instructions. The specific coding regions within the *PMFBP1* and *SUN5* genes were subsequently amplified using the obtained genomic DNA as a template. The PCR products were then detected via an ABI 3100 Genetic Analyzer (Applied Biosystems, Foster City, CA, USA), followed by Sanger sequencing to validate any mutations present in *PMFBP1* and Sad1 and *SUN5*.

### Western blotting analysis

Fresh semen was subjected to three rounds of centrifugation at 6000 rpm for 1 min in PBS. The resulting supernatant was discarded, and the precipitate was dissolved in loading buffer at a concentration of 1 mM at 100 °C for 10 min. The proteins were subsequently separated via SDS‒PAGE and transferred onto a nitrocellulose membrane by electroblotting at 100 V for an hour and a half at 4 °C. Prior to incubation with primary antibodies (anti-*PMFBP1* diluted to 1:1000 or anti-α-tubulin diluted to 1:1000; Sigma), the membrane was blocked with a solution containing BSA at room temperature for one hour. To detect the antigen content, secondary antibodies (goat anti-rabbit IgG or goat anti-mouse IgG diluted to 1:5000; ProteinTech Group, Hubei, China) were used.

### Immunofluorescence analysis

The sperm samples were subjected to immunofluorescence analysis following previously established protocols [9, 11]. The patient's sperm samples were first fixed onto glass slides. The fixed samples were then permeabilized to allow better antibody penetration into the cells. Next, the primary antibodies (4′, 6-diamidino-2-phenylindole(DAPI), anti-*PMFBP1* and anti-CD46 Thermo) were applied to the samples at a dilution of 1:200. After incubation with these primary antibodies, any unbound antibodies were washed away thoroughly. To detect the bound primary antibodies and visualize their respective target proteins within the sperm cells, secondary fluorescently labeled antibodies were employed.

### Minigene construction

Eligible DNA was used to amplify exons 13–16 and their short intronic flanking regions (Fig. 4A). The amplified fragments were subsequently inserted into the pcDNA3.1 vector by subcloning. Site-directed mutagenesis was performed via a mutagenesis kit to introduce the desired mutation according to the manufacturer's instructions. The primers used in this study are listed in Table 2, and all the constructs were verified via Sanger sequencing analysis.

**Table 2:** Primers used to construct minigenes

	F/R^a^	Primer Sequence (5’ to 3’)
**1**^**b**^	F	TTACAGGCGTTGGAGGAAAC
	R	AAGTAGAGCTGTAGGTGGCC
**2**^**c**^	F	GTTAACACGGACTCTGCAGG
	R	CGATCTTTGCTGCCTCTGTC
**3**^**d**^	F	AGCTCTACTTGTTAACACGGACTCTGCAGG
	R	CCGTGTTAACAAGTAGAGCTGTAGGTGGCC
**4**^**d**^	F	CTATAGGGAGACCCAAGCTTCACAGGGAGCAAGGCTCCA
	R	ACGTCATATGGATAGGATCCCTCCAGCTCACTCTCCTGGT
**5**^**e**^	F	ATCACGTGACCTCAGAGACAAAGAGCCTGCAGCAAA
	R	TCTGAGGTCACGTGATTGAGCTTCCGGAAAAGACAAAG
**6**^**f**^	F	AGACAAGACGTTGAAAGAG
	R	CGTGATTGAGCTTCTCGAG

^a^F represents the forward primer. R represents the reverse primer

^b^It was used to amplify exons 13–14 with complete intron 13 and partial intron 14

^c^Exons 15–16 with complete intron 15 and partial intron 14

^d^These sequences were used to connect two amplifications and construct wild-type minigenes

^e^Mutant minigenes were constructed

^f^It was used to determine the removal resulting from splice-site mutation

### Transfection

HEK293T cells, derived from human embryonic kidney cells, were cultured in Dulbecco's modified Eagle growth medium (DMEM, Invitrogen) supplemented with 10% fetal bovine serum (FBS, HyClone), 100 units/ml penicillin, and 100 μg/ml streptomycin (Invitrogen). The cells were maintained at 37 °C with a CO2 concentration of 5%. Next, the expression plasmids with wild-type and mutant minigenes were separately introduced into the cells through transfection. The final concentration of each transfected plasmid was 0.01 μg/μl. Finally, the cells were harvested after an additional period of 48 h.

### RT‒PCR and Sanger sequencing

After 48 h of transfection, the HEK-293 T cells were harvested, and total RNA was extracted via the TRIzol method. Subsequently, DNaseI treatment was performed to remove any contaminating DNA. Following that, 800–1000 ng of total RNA was reverse transcribed using the Revert Aid H Minus First Strand cDNA Synthesis Kit as per the manufacturer's instructions. PCRs amplification with specific primers targeting the partial *PMFBP1* transcript was then carried out. Finally, Sanger sequencing analysis was conducted on the resulting PCR products.

## Results

### Clinical findings

**Fig. 1:**
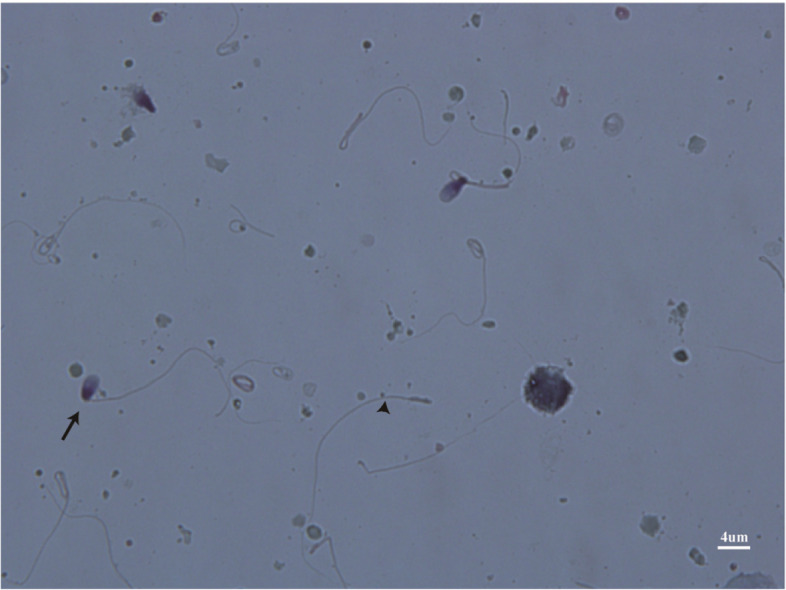
Papanicolaou staining of the spermatozoa from the patient. The sperm morphology analysis revealed a high prevalence of headless sperm tails (pinhead sperm) (black arrowhead), a limited quantity of tailless sperm heads, and aberrant connections between the head and tail segments (black arrow). 100 times oil microscope observation, scale bars = 4 µm

On the basis of routine semen analysis (Table 1) and abnormal sperm morphology (Fig. 1), the patient was diagnosed with ASS in accordance with previous studies. Notably, sperm morphology analysis revealed a high prevalence of headless sperm tails (pinhead sperm), a limited quantity of tailless sperm heads, and aberrant connections between the head and tail segments (Fig. 1). The patient's karyotype was determined to be 46; XY, and Y chromosome microdeletion analysis revealed no evident abnormalities. Color ultrasound examination confirmed that the bilateral testes were normal in size without any apparent varicose veins or prostate abnormalities, ruling out other potential causes contributing to infertility.

### Genetic findings

**Fig. 2:**
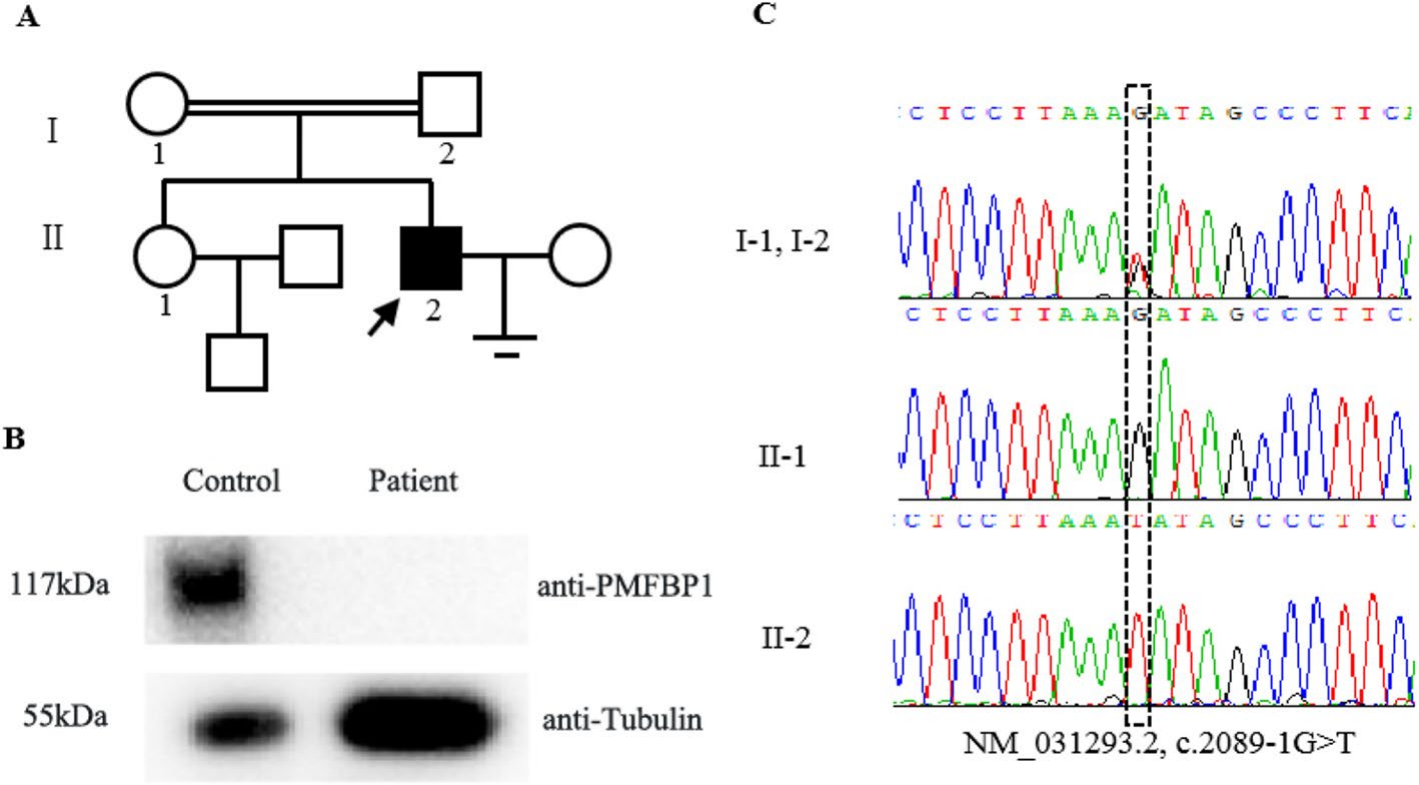
Pedigrees of a family with inherited *PMFBP1* mutations and protein expression of *PMFBP1* in the spermatozoa from the patient. **A** Proband (Π-2) has a homozygous splice site mutation in *PMFBP1*. **B** Western blotting was performed to test the expression level of *PMFBP1* in the control and patient sperm. Our findings revealed a complete absence of *PMFBP1* protein expression in patient sperm, whereas control samples presented detectable levels of the *PMFBP1* protein. **C** Sanger sequencing revealed a homozygous splice site mutation (NM_031293.2, c.2089-1G > T) in *PMFBP1* in the patient

To determine the causative factor of ASS in the patient, DNA was extracted from peripheral blood samples. Previous studies have established that *PMFBP1* and *SUN5* are the primary genes associated with ASS. Therefore, Sanger sequencing was performed to analyze the coding regions of *SUN5* and *PMFBP1* in the patient family(patient, patient parents and his sister). No mutations were detected in *SUN5*. However, Sanger sequencing revealed a homozygous splice site mutation (NM_031293.2, c.2089-1G > T) in *PMFBP1* in the patient (Fig. 2C). Furthermore, this splice site mutation was inherited from both parents (Fig. 2A).

### Western blotting detected the expression of *PMFBP1*

To evaluate the impact of splice site mutations on *PMFBP1* protein expression, we conducted western blot analysis to assess the presence of the *PMFBP1* protein in patient sperm and control samples. Our findings revealed a complete absence of *PMFBP1* protein expression in patient sperm, whereas control samples presented detectable levels of the *PMFBP1* protein (Fig. 2B).

### Immunofluorescence localization of the PMFBP1 protein

**Fig. 3:**
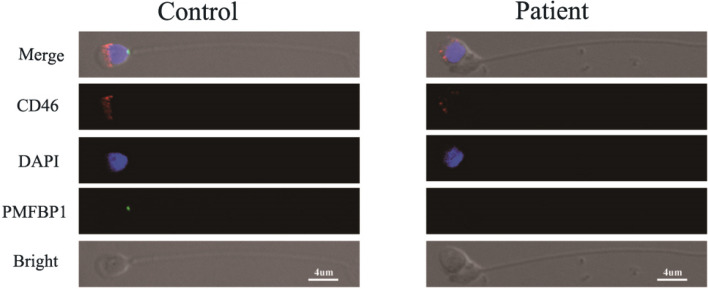
Immunofluorescence analysis of the spermatozoa from the control and from the patient. The acrosome, nucleus, and neck of the sperm were stained with CD46 (red), DAPI (blue) and *PMFBP1* antibodies (green), respectively. The localization of *PMFBP1* in sperm from control and patient samples was determined by immunofluorescence staining. The *PMFBP1* protein is localized at the head–tail junction of spermatozoa in control samples but is not detected in patient spermatozoa. 40 times oil microscope observation, scale bar = 4 µm

The localization of *PMFBP1* was determined through immunofluorescence analysis. Our findings indicate that the *PMFBP1* protein is localized at the head–tail junction of spermatozoa in control samples but is not detected in patient spermatozoa (Fig. 3). The fluorescence patterns observed for CD46 and DAPI, which are used to stain the acrosome and nucleus, respectively, were similar between the control and patient groups (Fig. 3). These results provide further evidence supporting the hypothesis that a splice-site mutation in *PMFBP1* leads to an absence of expression of this protein in patients.

### RT‒PCR and Sanger sequencing in vitro

**Fig. 4:**
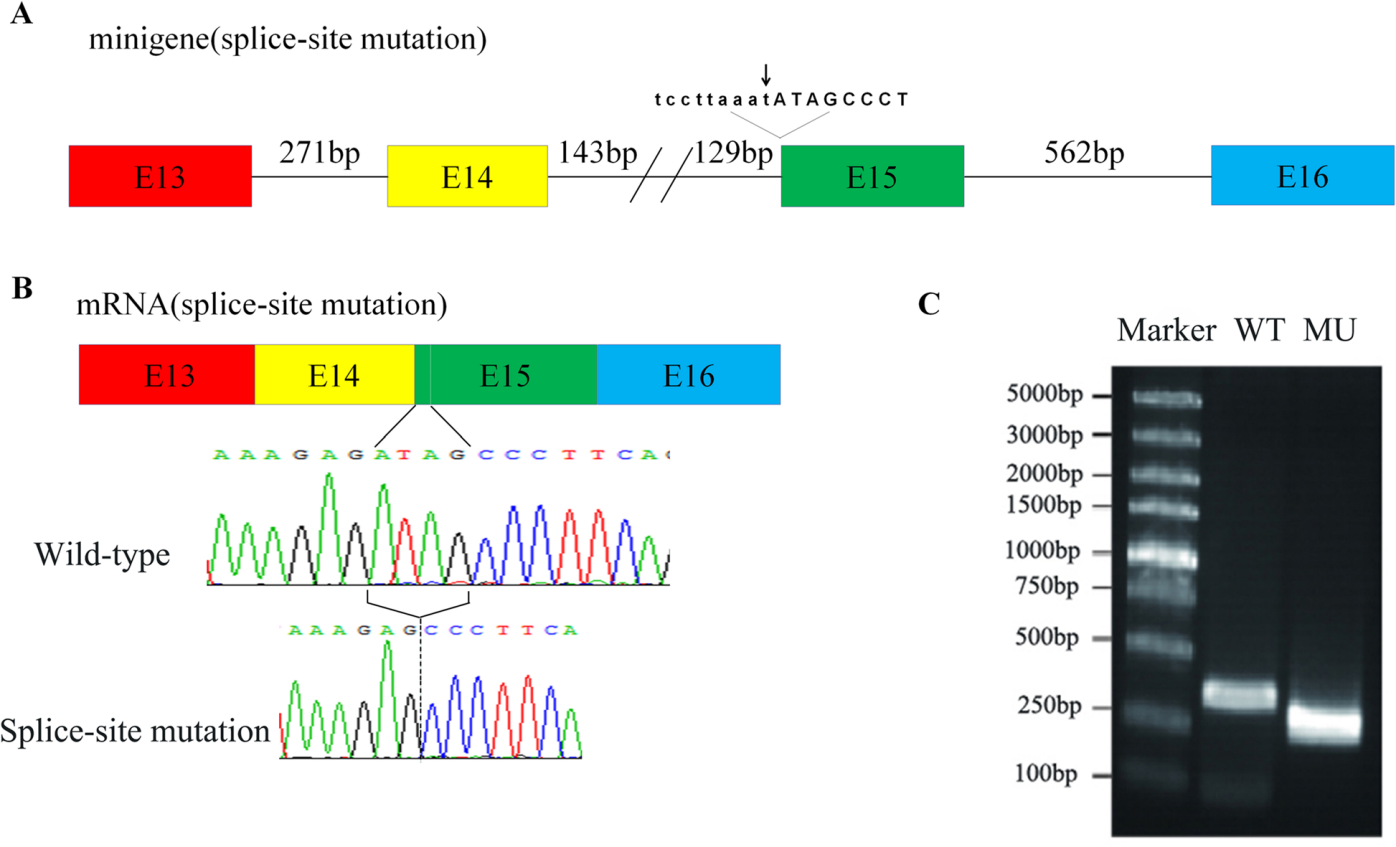
Determination of the removal after transcription. **A** The boxes with different colors represent the four exons. Minigenes were created using exons 13–16, complete introns 13 and 15, and two partial intron 14. The arrow indicates the mutation site. **B** Sanger sequencing confirmed that the splice-site mutation in *PMFBP1* led to a deletion of 4 base pairs within exon 15. **C** RT‒PCR analysis revealed a reduced transcriptional output for the splice-site mutant minigenes compared with their wild-type counterparts(WT:wild-type; MU:splice-site mutation)

To elucidate the underlying mechanism of the splice site mutation in *PMFBP1*, we transfected both wild-type and mutant minigenes into HEK-293 T cells. Subsequent RT‒PCR analysis revealed a reduced transcriptional output for the mutant minigenes compared with their wild-type counterparts (Fig. 4C). Furthermore, Sanger sequencing confirmed that the splice-site mutation in *PMFBP1* led to a deletion of 4 base pairs within exon 15 (Fig. 4B).

## Discussion

ASS is a rare and severe form of male infertility that poses significant challenges for affected individuals and their families [3–9]. In this study, we encountered a primary infertile patient who was diagnosed with ASS through semen analysis and Papanicolaou staining techniques. By performing Sanger sequencing on the DNA sample, we identified a homozygous splice-site mutation (2089-1G > T; NM_031293.2) within the *PMFBP1* gene as the likely pathogenic factor responsible for causing ASS in this particular patient.

According to previous studies, *PMFBP1* and *SUN5* mutations are primarily responsible for ASS [11–15, 18]. The *PMFBP1* gene is located on human chromosome 16 and consists of 27 exons. The coding region of the gene spans 3,024 bases and encodes a protein consisting of 1,007 amino acids [25]. *PMFBP1* is specifically expressed in both human and mouse testes [12, 26]. It localizes to the junction area between the head and flagella of sperm cells, and its expression is lost in patients carrying *PMFBP1* mutations [11, 12]. Functionally, *PMFBP1* interacts with *SUN5* and *SPATA6* to facilitate the connection between the head and tail of sperm cells. In male mice, mutations in *PMFBP1* disrupt this cooperation, leading to headless sperm. Therefore, *PMFBP1* mutation is believed to play a significant role in acephalospermia, which can cause male infertility [11, 12]. The separation in subtype II is positioned between the nucleus and the proximal centriole [27]. Reproductive success was observed in clinical pregnancies achieved through intracytoplasmic spermine injection (ICSI) methods in individuals with subtype II mutations within *PMFBP1* [26, 27].

In our study, we analyzed the protein expression of *PMFBP1* in patient samples compared with controls via western blotting. Our results revealed an absence of detectable levels of the *PMFBP1* protein in patient sperm, whereas control samples presented positive expression. These findings indicate that the splice site mutation leads to an absence of functional *PMFBP1* protein production. Immunofluorescence analysis further confirmed that the splice site mutation resulted in the absence of detectable levels of the *PMFPB1* protein.

Furthermore, to further investigate the functional consequences of the splice site mutation in *PMFBP1*, we constructed minigenes. These minigenes were then transfected into HEK-293 T cells. The results of our experiments revealed that this particular splice-site mutation led to a deletion of 4 base pairs in exon 15 when tested in vitro. This deletion ultimately resulted in an ASS phenotype, which is characterized by certain developmental abnormalities. On the basis of our observations, we hypothesized that this specific mutation introduces a premature stop codon after the translation of six amino acids (NM_031293.2, c.2089-1G > T, p.I697Pfs7*). Importantly, such mutations can have significant implications for protein structure and function. To confirm these findings and gain further insights into the effects of this mutation on mRNA splicing and protein production, we attempted RT‒PCR analysis using sperm mRNA from the studied patients. However, due to the limited availability of sperm heads from this individual, we were unable to extract sufficient sperm mRNA for successful analysis. Despite these limitations, our initial experiments with minigenes provided valuable information regarding the impact of this splice site mutation on *PMFBP1* expression. Further studies utilizing alternative approaches or larger sample sizes may be necessary to fully elucidate its role in disease development and progression.

### Limitations

Because ASS is rare, our study was limited by the small size of our sample, which can be considered the primary constraint of this study. However, given the rarity of our targeted population, a limited sample size was unavoidable. Another limitation of this study is the inability to obtain sufficient sperm heads from this individual. Furthermore, the inability to detect the expression of sperm mRNA was identified as an additional limitation. Finally, the pathogenesis of ASS caused by different mutation sites is different. However, the mechanism by which specific mutation sites lead to acephalospermia is well established.

## Conclusion

Our study identified a homozygous splice-site *PMFBP1* mutation in an ASS patient, which led to the removal of 4 base pairs in exon 15 and subsequently resulted in the absence of expression of the *PMFBP1* protein. Our findings add to the growing body of evidence supporting the involvement of *PMFBP1* in male infertility disorders such as ASS. Understanding how this mutation affects sperm development at both the cellular and molecular levels may provide valuable insights into potential therapeutic targets or interventions aimed at improving fertility outcomes for affected individuals.

## Data Availability

No datasets were generated or analysed during the current study.

## Abbreviations

*ACTRT1*: Actin-related protein T1
ASS: Acephalic spermatozoa syndrome
*BRDT*: Bromodomain testis associated
DAPI: 4′, 6-Diamidino-2-phenylindole
*DNAH6*: Dynein axonemal heavy chain 6
*HOOK1*: Hook microtubule tethering protein 1
ICSI: Intracytoplasmic sperm injection
HEK293T: Human embryonic kidney cells
*PMFBP1*: Polyamine modulated factor 1 binding protein 1
*SUN5*: Sad1 and UNC84 domain containing 5
*SPATC1L*: Spermatogenesis and centriole-associated 1 like
*SPATA20*: Spermatogenesis-associated gene 20
*SPATA6*: Spermatogenesis-associated gene 6
*TSGA10*: Testis-specific gene antigen 10

## References

[1] Inhorn MC, Patrizio P. Infertility around the globe: new thinking on gender, reproductive technologies and global movements in the 21st century. Hum Reprod Update. 2015;21(4):411–26. 10.1093/humupd/dmv016. 25801630 10.1093/humupd/dmv016

[2] Agarwal A, Mulgund A, Hamada A, Chyatte MR. A unique view on male infertility around the globe. Reprod Biol Endocrinol. 2015;13:37. 10.1186/s12958-015-0032-1. 25928197 10.1186/s12958-015-0032-1 PMC4424520

[3] Perotti ME, Giarola A, Gioria M. Ultrastructural study of the decapitated sperm defect in an infertile man. J Reprod Fertil. 1981;63(2):543–9. 10.1530/jrf.0.0630543. 7299757 10.1530/jrf.0.0630543

[4] Baccetti B, Selmi MG, Soldani P. Morphogenesis of ‘decapitated’ spermatozoa in a man. J Reprod Fertil. 1984;70(2):395–7. 10.1530/jrf.0.0700395. 6699806 10.1530/jrf.0.0700395

[5] Chemes HE, Puigdomenech ET, Carizza C, Olmedo SB, Zanchetti F, Hermes R. Acephalic spermatozoa and abnormal development of the head-neck attachment: a human syndrome of genetic origin. Hum Reprod. 1999;14(7):1811–8. 10.1093/humrep/14.7.1811. 10402395 10.1093/humrep/14.7.1811

[6] Toyama Y, Iwamoto T, Yajima M, Baba K, Yuasa S. Decapitated and decaudated spermatozoa in man, and pathogenesis based on the ultrastructure. Int J Androl. 2000;23(2):109–15. 10.1046/j.1365-2605.2000.t01-1-00217.x. 10762437 10.1046/j.1365-2605.2000.t01-1-00217.x

[7] Chemes HE, Carizza C, Scarinci F, Brugo S, Neuspiller N, Schwarsztein L. Lack of a head in human spermatozoa from sterile patients: a syndrome associated with impaired fertilization. Fertil Steril. 1987;47(2):310–6. 10.1016/s0015-0282(16)50011-9. 3545911 10.1016/s0015-0282(16)50011-9

[8] Baccetti B, Burrini AG, Collodel G, Magnano AR, Piomboni P, Renieri T, et al. Morphogenesis of the decapitated and decaudated sperm defect in two brothers. Gamete Res. 1989;23(2):181–8. 10.1002/mrd.1120230205. 2731903 10.1002/mrd.1120230205

[9] Porcu G, Mercier G, Boyer P, Achard V, Banet J, Vasserot M, et al. Pregnancies after ICSI using sperm with abnormal head-tail junction from two brothers: case report. Hum Reprod. 2003;18(3):562–7. 10.1093/humrep/deg121. 12615825 10.1093/humrep/deg121

[10] Li L, Sha YW, Xu X, Mei LB, Qiu PP, Ji ZY, et al. *DNAH6* is a novel candidate gene associated with sperm head anomaly. Andrologia. 2018;e12953. 10.1111/and.12953 10.1111/and.12953 29356036

[11] Sha YW, Wang X, Xu X, Ding L, Liu WS, Li P, et al. Biallelic mutations in *PMFBP1* cause acephalic spermatozoa. Clin Genet. 2019;95(2):277–86. 10.1111/cge.13461. 30298696 10.1111/cge.13461

[12] Zhu F, Liu C, Wang F, Yang X, Zhang J, Wu H. Mutations in *PMFBP1* cause acephalic spermatozoa syndrome. Am J Hum Genet. 2018;103(2):188–99. 10.1016/j.ajhg.2018.06.010. 30032984 10.1016/j.ajhg.2018.06.010 PMC6080767

[13] Zhu F, Wang F, Yang X, Zhang J, Wu H, Zhang Z. Biallelic *SUN5* mutations cause autosomal-recessive acephalic spermatozoa syndrome. Am J Hum Genet. 2016;99(4):942–9. 10.1016/j.ajhg.2016.08.004. 27640305 10.1016/j.ajhg.2016.08.004 PMC5065659

[14] Sha YW, Xu X, Ji ZY, Lin SB, Wang X, et al. Genetic contribution of *SUN5* mutations to acephalic spermatozoa in Fujian China. Gene. 2018;647:221–5. 10.1016/j.gene.2018.01.035. 29331481 10.1016/j.gene.2018.01.035

[15] Fang J, Zhang J, Zhu F, Yang X, Cui Y, Liu J. Patients with acephalic spermatozoa syndrome linked to *SUN5* mutations have a favorable pregnancy outcome from ICSI. Hum Reprod. 2018;33(3):372–7. 10.1093/humrep/dex382. 29329387 10.1093/humrep/dex382

[16] Sha YW, Sha YK, Ji ZY, Mei LB, Ding L, et al. *TSGA10* is a recurrent candidate gene associated with acephalic spermatozoa. Clin Genet. 2018;93(4):776–83. 10.1111/cge.13140. 28905369 10.1111/cge.13140

[17] Ye Y, Wei X, Sha Y, Li N, Yan X, Cheng L, et al. Loss-of-function mutation in *TSGA10* causes acephalic spermatozoa phenotype in human. Mol Genet Genomic Med. 2020;8(7):e1284. 10.1002/mgg3.1284. 32410354 10.1002/mgg3.1284 PMC7336754

[18] Liu G, Wang N, Zhang H, Yin S, Dai H, Lin G, Li WN. Recurrent mutations in *PMFBP1*, *TSGA10* and *SUN5*: expanding the spectrum of mutations that may cause acephalic spermatozoa. Clin Genet. 2020;97(6):938–9. 10.1111/cge.13747. 32285443 10.1111/cge.13747

[19] Li L, Sha Y, Wang X, Li P, Wang J, Kee K, Wang BB. Whole-exome sequencing identified a homozygous *BRDT* mutation in a patient with acephalic spermatozoa. Oncotarget. 2017;8(12):19914–22. 10.18632/oncotarget.15251. 28199965 10.18632/oncotarget.15251 PMC5386733

[20] Chen H, Zhu Y, Zhu Z, Zhi E, Lu K, Wang X, et al. Detection of heterozygous mutation in hook microtubule-tethering protein 1 in three patients with decapitated and decaudated spermatozoa syndrome. J Med Genet. 2018;55(3):150–7. 10.1136/jmedgenet-2016-104404. 29330334 10.1136/jmedgenet-2016-104404

[21] Sha Y, Liu W, Li L, et al. Pathogenic variants in *ACTRT1* cause acephalic spermatozoa syndrome. Front Cell Dev Biol. 2021;9:676246. 10.3389/fcell.2021.676246. 34422805 10.3389/fcell.2021.676246 PMC8377740

[22] Li YZ, Li N, Liu WS, et al. Biallelic mutations in spermatogenesis and centriole-associated 1 like (*SPATC1L*) cause acephalic spermatozoa syndrome and male infertility. Asian J Androl. 2021;23:1–6. 10.4103/aja.aja5621. 34213489 10.4103/aja.aja_56_21 PMC8788604

[23] Wang X, Jiang C, Dai S, et al. Identification of nonfunctional *SPATA20* causing acephalic spermatozoa syndrome in humans. Clin Genet. 2023;103(3):310–9. 10.1111/cge.14268. 36415156 10.1111/cge.14268

[24] World Health Organization. WHo laboratory manual for the examination and processing of human semen[M]. 16th ed. Geneva: WHO Press; 2121.

[25] Wu C, Jin X, Tsueng G, et al. BioGPS: Building your own mash-up of gene annotations and expression profiles. Nucleic Acids Res. 2016;44(D1):D313–6. 10.1093/nar/gkv1104. 26578587 10.1093/nar/gkv1104 PMC4702805

[26] Ohuchi J, Arai T, Kon Y, Asano A, Yamauchi H, Watanabe T. Characterization of a recurrent gene sperm-tail-associated protein (Stap) in mouse post-meiotic testicular germ cells. Mol Reprod Dev. 2001;59(4):350–8. 10.1002/mrd.1041. 11468771 10.1002/mrd.1041

[27] Nie H, Tang YG, Qin WB. Beyond Acephalic Spermatozoa: The Complexity of Intracytoplasmic Sperm Injection Outcomes. Biomed Res Int. 2020;2020:6279795. 10.1155/2020/6279795. 32104701 10.1155/2020/6279795 PMC7035536

